# Laparoscopic ovarian transposition before pelvic radiation in rectal cancer patient: safety and feasibility

**DOI:** 10.1186/1750-1164-6-9

**Published:** 2012-09-17

**Authors:** Sami Al-Asari, Alaa Abduljabbar

**Affiliations:** 1 Department of colorectal surgery, King Faisal Specialist Hospital and Research Centre, Riyadh, Saudi Arabia

**Keywords:** Laparoscopic, Ovarian, Transposition, Rectal cancer

## Abstract

**Background:**

Infertility due to pelvic radiation for advanced rectal cancer treatment is a major concern particularly in young patients. Pre-radiation laparoscopic ovarian transposition may offer preservation of ovarian function during the treatment however its use is limited.

**Aim:**

The study investigates the safety, feasibility and effectiveness of pre-radiation laparoscopic ovarian transposition and its effect on ovarian function in the treatment o locally advanced rectal cancer.

**Methods:**

Charts review of all young female patients diagnosed with locally advanced rectal cancer, underwent laparoscopic ovarian transposition, then received preoperative radiotherapy at king Faisal Specialist Hospital and Research Centre between 2003–2007.

**Results:**

During the period studied three single patients age between 21–27 years underwent pre-radiation laparoscopic ovarian transposition for advanced rectal cancer. All required pretreatment laparoscopic diversion stoma due to rectal stricture secondary to tumor that was performed at the same time. One patient died of metastatic disease during treatment. The ovarian hormonal levels (FSH and LH) were normal in two patients. One has had normal menstrual period and other had amenorrhoea after 4 months follow-up however her ovarian hormonal level were within normal limits.

**Conclusions:**

Laparoscopic ovarian transposition before pelvic radiation in advanced rectal cancer treatment is an effective and feasible way of preservation of ovarian function in young patients at risk of radiotherapy induced ovarian failure. However, this procedure is still under used and it is advisable to discuss and propose it to suitable patients.

## Introduction

Colorectal cancer is the third most common cancer in the world and has significant mortality. Rectal cancer is usually staged with modalities such as endorectal ultrasound, Magnetic Resonance Imaging (MRI) and Computed Tomography (CT) of chest abdomen and pelvic.

Neoadjuvant chemo-radiation therapy is used routinely in low-lying rectal tumor to reduced local and systemic recurrence, improve respectability and attempt sphincter preservation [1,2].

Pelvic radiation and chemotherapy particularly in the young female may cause infertility. Preservation of ovarian function by transposition of ovaries before pelvic radiation has been suggested in all reproductive women [3]. This procedure has been frequently offer to patients with advanced cervical cancer and Hodgkin’s lymphoma having chemo-radiotherapy but less popular in advanced rectal cancer.

The estimated dose of radiation at which half of the follicles are lost in humans (LD50) is 4Grays [4]. Women less than 40 years old are less sensitive to radiation induced ovarian injury. However permanent ovarian failure will occur on exposure of 20Grays or over of pelvic radiation while old patients only require 6Grays for ovarian failure [5]. In the ovarian transposition procedure both ovaries relocate temporally away from the direct radiation area. The ovarian transposition will prevent direct radiation induced injury to gynecological apparatus and helps in prevention of premature menopause. Although the procedure is well established as part of treatment in other malignancies little is known about its safety and feasibility in advanced rectal cancer.

## Aim

The study investigates the safety, feasibility and effectiveness of pre-radiation laparoscopic ovarian transposition and its effect on ovarian function in the treatment of locally advanced rectal cancer.

## Methods

Retrospective data were collected of all three patients underwent laparoscopic ovarian transposition before long course chemo-radiotherapy for locally advanced rectal cancer. The procedures were carried out at King Faisal Specialist Hospital and Research Centre in Saudi Arabia during 2003 to 2007.

A detailed history was taken from all three patients including their menstruation cycle and previous gynecological treatment. Blood were taken for hematological and biochemical makers including Follicle stimulating hormone (FSH) and Luteinising hormone (LH). All blood markers were measure before treatment and at 1, 2,4 and 6 months post-treatment.

Technically, we perform the ovarian transposition using minimal access approach in all three cases. The procedures were carried out under general anesthesia and a Foley’s catheter was placed to empty the bladder. The patient was placed in Trendelenburg’s position and a 12 mm umbilical port inserted for abdominal cavity access using open technique. Insufflation was achieved using carbon dioxide (CO2) gas and abdominal pressure of 12 mm of Hg was maintained during the procedure. A 5 mm port was inserted under vision in right and another one at left lumber region. The umbilical port was used for the 10 mm 300 angled camera and 5 mm ports used as working ports. A diagnostic laparoscopy was performed to assess the tumor stage. Then, the peritoneum over pelvic sidewall was mobilized to develop a retroperitoneal space. All major vasculature (common, external and internal Iliac) structure were identified and preserved. The ovarian vessels and both ureters were exposed along its whole length. A Harmonic scalpel used to dissect the utero-ovarian ligaments. The ovarian vessel was carefully mobilized to make sure the blood supply to the structure was not compromised. Both ovaries and fallopian tubes were fixed at the lateral abdominal wall below the spleen on the left and liver on the right using Titanium based tuckers (ProTacks). A 5 mm metallic clip was placed at the base of both ovaries as a marker.

**Figure 1:**
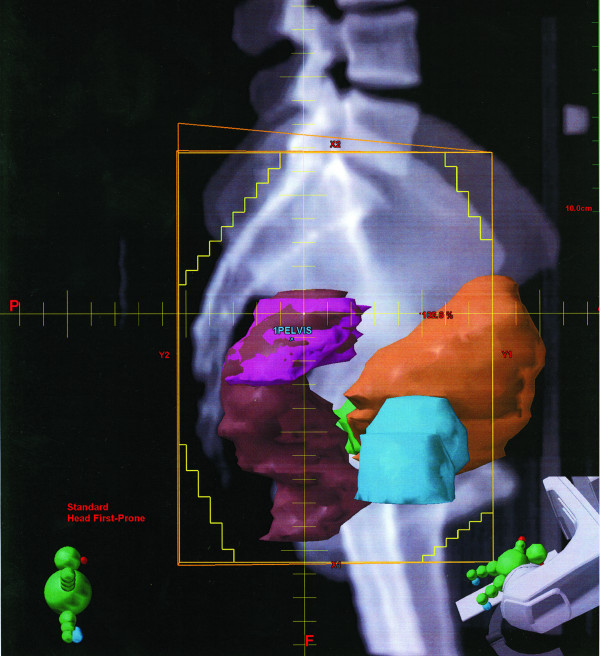
Lateral view, pink color is the targeted tumor area.

**Figure 2:**
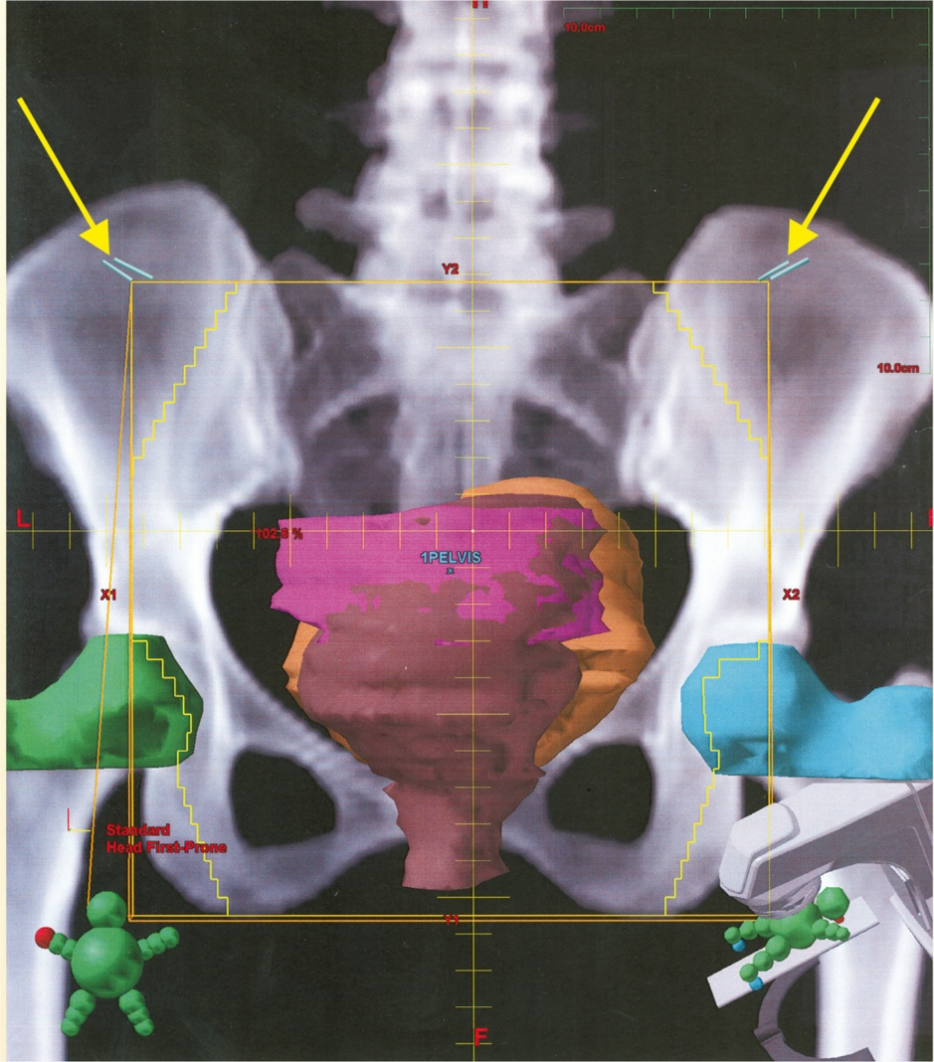
A-P View, Bilateral yellow arrows pointing on the clips out side the radiation therapy field.

**Figure 3:**
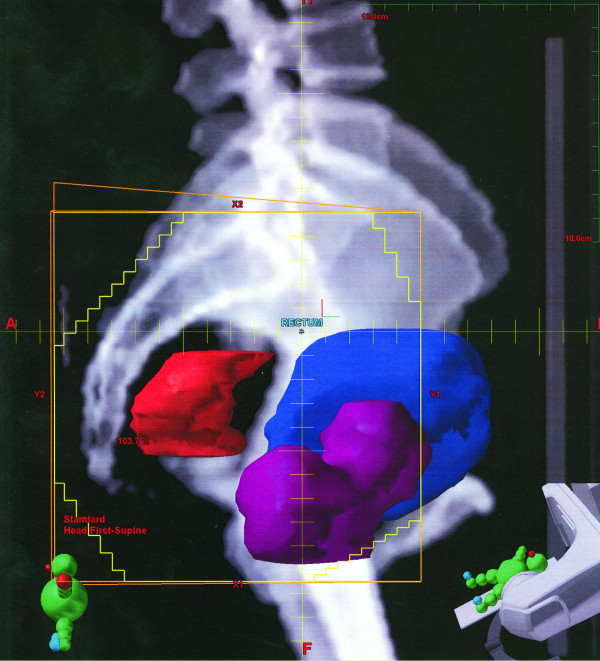
Lateral view of the tumor (red).

**Figure 4:**
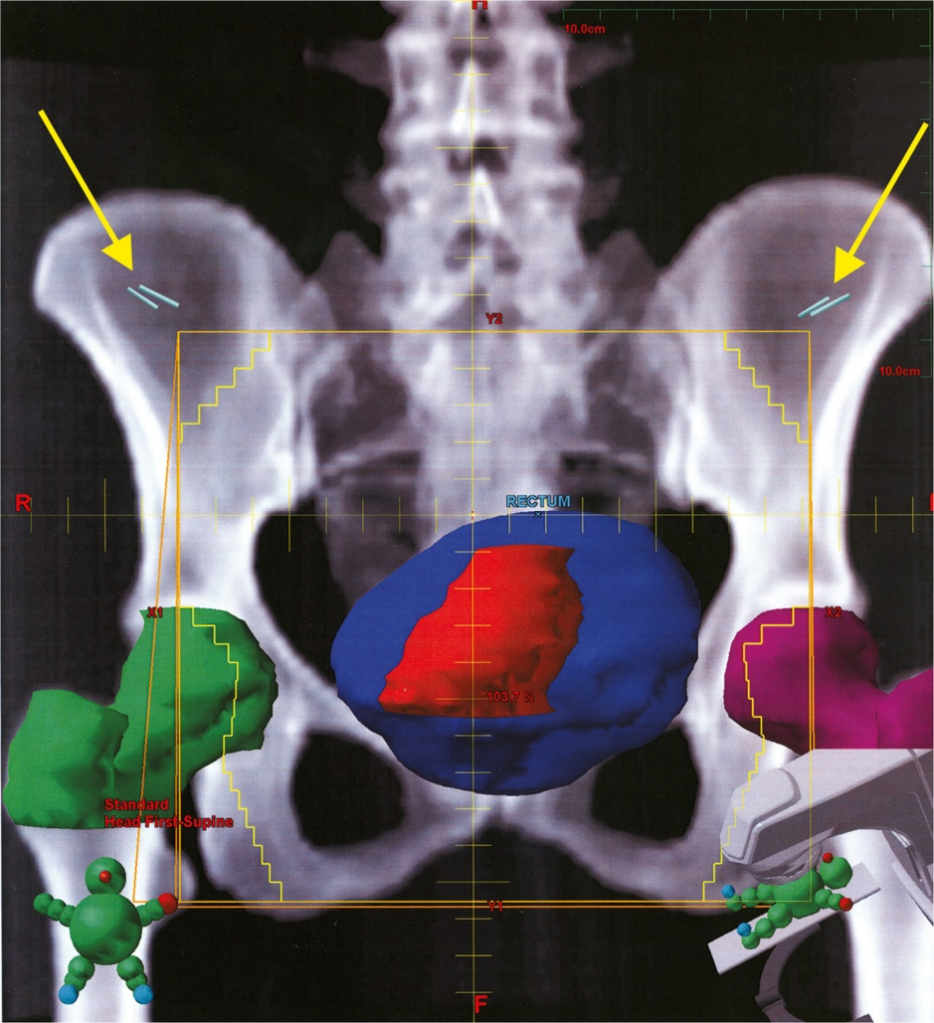
A-P view shows the site of the tumor (red) and the ovarian clips (blue).

The pre-radiation film confirmed the metallic clip outside the area of pelvic radiation in all cases. A laparoscopic diversion transverse colostomy was created at the pre-marked site at right side of abdomen to prevent complete large bowel obstruction during the rectal cancer treatment. Simulation of radiation therapy and the position of the clips demonstrated on (Figure 1,2,3, and 4).

## Results

We record 3 patients with age between 21–27 diagnosed with CT scan, MRI, Endorectal US, and colonoscopy to have a moderate differentiated rectal adenocarcinoma 4–10 cm from anal verge with compromised lumen. All of them underwent laparoscopic ovarian transposition and diverting colostomy. Postoperatively they received long course chemoradiation. All patients are single. The hormonal level were measured at 1,2,4 and 6 months and we found that the FSH range between (2–79.9 Iu/L) and LH (3.7- 36.2 Iu/L) which is within normal limit. There were no postoperative surgical related complications. On follow up, one patient died form neutropenic bowel perforation and sepsis and we found that she have liver metastasis during therapy, while the other tow maintain their menstruation with delay in one of them for four months.

## Discussion

Our study investigated the safety and feasibility of the laparoscopic ovarian transposition before pelvic radiation in advanced rectal cancer treatment. This is usually measured by quantitative analysis of ovarian hormones as well as fertility outcome [3].

Spontaneous pregnancies are possible if tubal function is preserved as part of the ovarian transposition and fixation [3].

Morice et al. [5] reported on 37 consecutive cases of ovarian transposition procedures. In his study 16 patients become pregnant spontaneously 12 of which did not have the ovaries repositioned from the adnexa. Similarly, Dabirashrafi et al. have reported intrauterine pregnancy after laparoscopic ovarian transposition in another study [6].

Young patients require long course chemo-radiotherapy for advanced rectal cancer should be offer laparoscopic ovarian transposition before the radiation in which preservation of ovarian function is desired. We believe that placement of metallic clips at the base of ovaries as maker help the radiotherapist to identify this structure before radiation. The laparoscopic ovarian transposition preserved the ovarian hormonal function after the rectal cancer treatment in our patients. However none of them so far conceive, therefore impact of the procedure on fertility requires longer follow-up.

The laparoscopic procedure has been reported for different pelvic pathology and achieves almost a comparable successes rate.

Literature review for the cervical and anorectal cancer cases were they had the same procedure with different success rate and the length of the follow up summarized in Table 1. The infertility has a major impact on the quality of life of young female patients with advanced rectal cancer after treatment. Our study suggests that laparoscopic ovarian transposition is safe, effective and feasible in preservation of ovarian hormonal function during and after the pelvic radiation therapy in advanced rectal cancer patients. However, further studies are required to assess its role in ovarian preservation and fertility before it could be widely used.

**Table 1:** Literature review of cervical and anorectal cancer

**Reference**	**Number**	**Treatment**	**Proportion of patients with preserved ovarian function**	**Follow-up (Months)**
Leonard A Farber [3]	1	Radiotherapy + LOT+LAR	100%	13
Hodel et al. [7]	9	RH+LOT±XRT	78%	Ns
Husseinzadeh et al. [8]	22	RH+LOT	100%	Ns
	14	LOT+XRT	67%	
	4	RH+LOT+XRT	50%	
Chambers et al. [9]	54	RH	96%	54(median)
	25	RH+LOT	96%	36(median)
Chambers et al. [10]	14	RH+LOT+ Radiotherapy	71%	35(median)
Feeney et al. [11]	58	RH+BSO±XRT	0%	24(mean)
	104	RH+LOT	97%	
	28	RH+LOT+XRT	50%	
Covens et al. [12]	3	PLND+LOT+XRT	66%	25-32
Van Eijkeren et al. [13]	36	RH+LOT	95%	36-48
	18	RH+LOT+XRT	72%	
Morice et al. [14]	11	RH+LOT	100%	31(median)
	59	RH+LOT+Brachy	90%	
	35	RH+LOT+XRT+Brachy	60%	
Yamamoto et al. [15]	30	RH+LOT	97/89%	At 12 v.s. At 60
	26	RH+LOT+XRT	65/39%	
Buekers et al. [16]	17	Radiotherapy + LOT ±RH	41%	At 12
Treissman MJ et al. [17]	1	Radiotherapy +LOT	50%	8

RH radical hysterectomy, LOT lateral ovarian transposition, XRT external beam radiotherapy, BSO bilateral salpingo-oophorectomy, PLND pelvic lymph node dissection only, Brachy brachytherapy, Ns not stated.

## Competing interest

The authors declare that they have no competing interests.

## Authors’ contribution

SA-A: Data collection, analysis and writing the manuscript. AA: Data collection and manuscript revision. Both authors read and approved the final manuscript.

## Funding

The funding for this article belong the author SAMI ALASARI.
